# Associations between psychosocial work environments and social capital: a multilevel analysis study in a Chinese context

**DOI:** 10.1186/s12889-018-5916-5

**Published:** 2018-08-06

**Authors:** Junling Gao, Jing Wang, Denglai Yu, Junming Dai, Yongkai Zhu, Hua Fu

**Affiliations:** 1 School of Public Health, Fudan University; Fudan Health Communication Institute, PO Box 248, 138 Yixueyuan Road, Shanghai, 200032 China; 2 Pudong Center of Disease Control and Prevention, Shanghai, 200136 China

## Abstract

**Background:**

Understanding the determinants of social capital is the prerequisite to building social capital. However there was few studies to explore factors related to workplace social capital. We aim to examine associations between psychosocial work environments and social capital in a Chinese context through a cross-sectional study.

**Methods:**

A cross-sectional study was conducted in Shanghai, China from December 2016 through March 2017. In total, 2380 workers from 32 workplaces were randomly sampled by a two-stage sampling procedure. Workplace social capital (WSC), psychosocial work environments (PWEs), and workplace Chinese Confucian values (CCVs), were assessed using validated and psychometrically tested measures. Multilevel ordinal regression models were used to examine the associations of WSC with individual- and workplace-level PWEs and workplace CCVs after controlling for individual socioeconomic characteristics.

**Results:**

After controlling for individual socioeconomic characteristics, all individual-level PWEs (unstandardized coefficients [B] ranging from 0.280 to 2.467) were positively associated with WSC. Individual-level workplace CCVs had mixed associations with WSC—high individual levels of respect for authorities (B: 0.325; 95%CI: 0.134, 0.516) and altruism (B: 0.347; 95%CI: 0.155, 0.539) were associated with high WSC, while high individual levels of acceptance of authorities (B: − 0.214; 95%CI: − 0.381, − 0.046) and the *mianzi* rule (B: − 0.258; 95%CI: − 0.435, − 0.080) were associatecd with low WSC. No workplace-level variable was associated with WSC.

**Conclusion:**

These findings suggest that workplace social capital associates with multiple factors. Psychosocial work environments and cultural context are important in understanding variations in workplace social capital between individuals.

## Background

Social capital is defined as features of social organization—including networks, norms, and social trust—that facilitate coordination and cooperation for mutual benefit [1]. In the public health literature, social capital is viewed as a feature not only of social context but also of the individual actors living within the social context [2] and has always been measured at both the micro- (individual) and macro-level (community, workplace) in empirical studies. Long working hours have become the culture of many workplaces [3], where people are typically exposed to a reasonable amount of social relationships and day-to-day interactions. Thus, the workplace might be a dominant resource for social capital compared with the neighborhood and community. Workplace social capital (WSC) was defined as the shared values, attitudes, and norms of trust and reciprocity as well as practices of collective action in workplace [4]. WSC could affect health via processes of informal social control, social cohesion, maintenance of healthy norms and reinforcing healthy behaviours, and the provision of access to social support [4, 5]. A growing body of research has shown that low WSC is associated with unhealthy behaviors [6–11], poor mental health [12–15], and high mortality [16].

Researchers [17, 18] have suggested building social capital as a strategy for health promotion within and between populations. However, there have been relatively few public health interventions that have actually targeted the social capital of individuals or populations to improve health [3], partly due to a lack of understanding of factors determining the levels of social capital.

Evidence has also found that both individual and macro factors are associated with social capital. At the individual level, previous studies found that age [19, 20], gender [20], educational attainment [19–21], marital status [20, 22], income [23, 24], and employment status [19, 23] were associated with social capital. However, the findings were not consistent; for example, Hanibuchi et al. [24] found that females showed lower general trust, while [20] found no gender differences in general trust. Moreover, macro-level variations in levels of social capital still exist after controlling for individual-level characteristics [20, 22, 24–26]. It is therefore necessary to examine the individual-level and macro-level determinants of social capital via multilevel study. Recently, several studies have also shown complex and contradictory associations between macro-level factors, including population density [27, 28], residential mobility [22], neighborhood walkability [24, 29–31], and streetscape greenery [32], and the level of social capital. Focus the interesting of current study, limited evidence has indicated that after controlling for individual characteristics, WSC was associated with work environment factors [33] such as, workplace demographics, employment patterns, work-unit size, psychosocial work environments [34–37], such as leadership, organizational justice, including job strain and effort/reward imbalance. Oksanen, et al. found that smaller work-unit size and higher turnover were associated with higher WSC [33].

In addition, social capital may be influenced by cultural background [20]. Chinese Confucian values(CCVs), the common base of social culture in China is a complex system of moral, social, political, and religious thoughts with regard to an individual’s relationships with others and appropriate conduct [38]. A cross-culture study indicated that the Chinese have highest level of collectivism and interpersonal trust among 42 countries [39] The concept most closely related to social capital is *guanxi* in Chinese culture, which refers to personal relationships, connections, or networks based on CCVs, which can potentially be utilized to acquire resources in informal and interpersonal forms [40]. These CCCs include *xinyong* (trustworthiness), *mianzi* (face), *renqing* (norms of interpersonal behavior), reciprocity, and obligation [41], which permeate all aspects of daily life in China. Theoretically, CCVs are distinctively about interpersonal relationships, while social capital is considered as both the attributes of individuals and organizations [42], so CCVs are different from social capital, but they are correlated; however, this has not been examined by formal study. Based on literature review, we hypothesize that WSC is associated with psychosocial work environment, CCVs after controlling for employee characteristics in Chinese workplace, and examine this hypothesis by a multilevel cross-sectional study.

### Study design and population

#### Study population

We conducted a cross-sectional study in Shanghai, China, from December 2016 to March 2017. In total, 2380 workers were randomly sampled through a two-stage sampling procedure. First, we selected 6 small workplaces (staff number < 100), 18 medium workplaces (100 ≤ staff number < 300), and 10 large workplaces (staff number ≥ 100) from the Pudong district of Shanghai using the convenience sampling method. In the second stage, we randomly sampled 100 workers from medium and large workplaces selected in the first stage; all workers from small workplace were included. Among 34 workplaces, there were 18 manufacturing enterprises, 8 service enterprises, 4 transport companies, 2 scientific institutions and 2 IT companies. A self-administered questionnaire was distributed via the Human Resources Department to all selected workers, who were provided written informed consents before filling in the questionnaires. Completion of the questionnaires was anonymous. The Institutional Review Board of the School of Public Health, Fudan University, approved the study.

#### Measures

##### Workplace social capital

Workplace social capital was assessed with the validated Chinese version [43] of the WSC scale, which consists of eight 5-point Liker-scale items (from 1 to 5). Cronbach’s alpha was 0.88 for the original scale and 0.95 for the current sample, indicating that the scale was acceptable. The items were as follows: (i) “We have a ‘we are together’ attitude”; (ii) “People feel understood and accepted by each other”; (iii) “People keep each other informed about work-related issues in the work unit”; (iv) “Members of the work unit build on each other’s ideas in order to achieve the best possible outcome”; (v) “People in the work unit cooperate in order to help develop and apply new ideas”; (vi) “We can trust our supervisor”; (vii) “Our supervisor treats us with kindness and consideration”; and (viii) “Our supervisor shows concern for our rights as an employee.” The mean score of the eight ratings was used to assess individual WSC. Higher score indicates higher level of individual WSC.

##### Individual characteristics

Besides gender, age (10-year categories), and marital status divided into married and unmarried(including divorce, widowed and single), we selected the following characteristics as indicators of socioeconomic status (SES): educational level (junior high school, senior high school, junior college, and college or higher), annual salary (< 50,000 yuan, 50,000–100,000 yuan, and ≥ 100,000 yuan), years in the workplace (2-year categories), and position (junior, intermediate, senior).

##### Psychosocial work environments

Psychosocial work environments (PWEs) pertain to interpersonal and social interactions that influence behavior and development in the workplace [44]. The second short version of the Copenhagen Psychosocial Questionnaire (COPSOQ II) [45] was used to assess the following aspects of psychosocial work environment: (i) Work organization and job content, consisting of eight items scored from 1 to 5 to assess personal influence and possibilities for development. Cronbach’s alpha was 0.91 for the present sample. (ii) Interpersonal relationships and leadership, consisting of 12 items scored from 1 to 5 to assess predictability, role clarity and conflicts, and quality of leadership. Cronbach’s alpha was 0.94 for the present sample. (iii) Work-life balance, consisting of three items scored from 1 to 4 to assess job satisfaction and work-family conflict. Cronbach’s alpha was 0.74 for the present sample. (iv) Workplace values, consisting of four items scored from 1 to 5 to assess trust regarding management and justice. Cronbach’s alpha was 0.89 for the present sample. The mean scores of the corresponding items were used to assess PWEs. Higher score indicates the better psychosocial work environment, for example, the higher score of work-life balance indicates more balanced work-life.

##### Workplace Chinese confucian values

In the current study, we focused on workplace Chinese Confucian values, which pertain to social norms with regards to building harmonious relationships at workplace. The Workplace Confucian Traditional Values scale [46] was used to assess four dimensions of workplace CCVs: respect for authorities (RA), altruism (AL), acceptance of authorities (AA), and the *mianzi* rule (MR). (i) RA was measured by the mean score of individual response to the following 6-point Likert items (from 1 = strongly disagree to 6 = strongly agree): “Following the advice of your elders is one of the best ways to avoid mistakes”; “When a dispute cannot be resolved, it should be done by the oldest colleague”; “A leader is like the head of the family, and the important thing in a company is to listen to his instructions”; “It is a virtue to respect and obey the leader.” Cronbach’s alpha was 0.84 for the present sample. (ii) AL was measured by the mean score of individual responses to the following 6-point Likert items (from 1 = strongly disagree to 6 = strongly agree): “All requests of the department and company should be accepted unconditionally”; “Even if others are wrong, I should try my best to tolerate and forgive them”; “I should try not to inconvenience others, even if I was inconvenienced”; “While it is not good for yourself, it is always good to help others”; “Personal interests should be subordinated to the interests of the department and company.” Cronbach’s alpha was 0.81 for the present sample. (iii) AA was measured by the mean score of individual response to the following 6-point Likert items (from 1 = strongly disagree to 6 = strongly agree): “Employees should obey even the company rules that are wrong”; “An employee’s salary should be firstly based on seniority, secondly based on individual ability”; “Employees should obey the rules and regulations of the company and not bother to understand the reason why they were established.” Cronbach’s alpha was 0.86 for the present sample. (iv) MR was measured by the mean score of individual responses to the following 6-point Likert items (from 1 = strongly disagree to 6 = strongly agree): “Mistakes are discussed alone, and virtues are stated openly”; “As long as the *mianzi* is passable, it’s nothing to take a small loss”; “Even if you have conflicts of interest with your colleagues, you cannot easily tear *mianzi.*” Cronbach’s alpha was 0.80 for the present sample. Higher scores indicate higher level of individual perception of workplace CCVs.

All the above PWEs and workplace CCVs were assessed in two ways: (a) at the individual level, using each individual’s own assessment; (b) at the aggregate level, summing up the assessments of all respondents from the same workplace. Both individual-level and aggregate-level PWEs and workplace CCVs were dichotomized into low and high by the median.

#### Statistical analyses

Descriptive statistics were performed firstly. Categorical variables were expressed as percentages and continuous variables were expressed as mean (95%CI). Because the WSC scores (scored from 1 to 5) did not comprise a normal distribution, Mann-Whitney Tests for two groups’ comparison and Kruskal-Wallis Tests for three or more groups’ comparison were used. Literature reviewed indicated that macro-level variations in levels of social capital still exist after controlling for individual-level characteristics. It is therefore necessary to examine the individual-level and macro-level determinants of social capital via multilevel analysis. Our data had a multilevel structure consisting of employees (at level 1) nested within workplaces (at level 2), and the WSC scores (scored from 1 to 5) did not comprise a normal distribution, so we used multilevel ordinal regression models to examine the relationships of WSC with individual- and workplace-level variables. Individual-level variables were individual socioeconomic characteristics, individual-level PWEs, and individual-level workplace CCVs. Workplace-level variables were aggregate-level PEWs and workplace CCVs as well as workplace size (small, medium, or large). The analysis was conducted in following steps: Firstly, we examine the workplace-level variance in WSC without including any explanatory variables (not shown in Table 2); next, we examined the relationships of WSC with workplace-level variables (model 1); thirdly, we examined the relationships of WSC with individual-level variables (model 2); finally, we modeled all individual and workplace-level variables simultaneously (model 3). R version 3.3.3 [47] was used for all analyses. The multi-level analyses were performed using the lme4 package [48].

## Results

As shown in Table 1, of the 2380 participants, more than half (51.3%) were female, and most (79.1%) were married. More than 60% (67.5%) were aged < 40 years old. About one quarter graduated from junior high school, and 22.6% graduated from college. More than half worked in medium workplaces; 11.9% had worked in the same workplace for less than 2 years, and 36.6% had worked in the same workplace for at least 10 years. The annual salary of 10% of participants was ≥100,000 yuan, while that of 46.8% was < 50,000 yuan. Only 5.1% of participants were in senior positions; most (68.6%) held junior positions.

**Table 1:** Comparisons of workplace social capital among demographic characteristics by univariate analysis

	*n* (%)	Mean (95%CI)	*p*
Gender			
Male	1,158 (48.7)	3.90 (3.86, 3.94)	0.7595
Female	1,222 (51.3)	3.90 (3.87, 3.94)	
Age			
< 30	654 (27.5)	3.96 (3.91, 4.01)	0.0120
30–39	955 (40.1)	3.92 (3.88, 3.96)	
40–49	533 (22.4)	3.82 (3.77, 3.88)	
≥ 50	238 (10.0)	3.85 (3.76, 3.94)	
Educational level			
Junior high school	587 (24.7)	3.88 (3.83, 3.93)	0.5621
Senior high school	724 (30.4)	3.88 (3.83, 3.93)	
Junior college	530 (22.3)	3.91 (3.86, 3.97)	
College or higher	539 (22.6)	3.94 (3.89, 3.99)	
Marriage status			
Married	1,883 (79.1)	3.93 (3.87, 3.98)	0.1913
Unmarried	497 (20.9)	3.89 (3.87, 3.92)	
Annual salary (thousand yuan)			
< 50	1,114 (46.8)	3.89 (3.85, 3.93)	0.7941
50–99	1,027 (43.2)	3.92 (3.88, 3.95)	
≥ 100	239 (10.0)	3.89 (3.82, 3.97)	
Working years			
< 2	283 (11.9)	4.07 (3.99, 4.14)	< 0.001
2–3	475 (20.0)	3.92 (3.86, 3.97)	
4–5	321 (13.5)	3.94 (3.88, 4.00)	
6–7	216 (9.1)	3.97 (3.89, 4.06)	
8–9	213 (8.9)	3.85 (3.76, 3.93)	
≥ 10	872 (36.6)	3.82 (3.78, 3.87)	
Position			
Senior	121 (5.1)	3.86 (3.73, 3.40)	0.1496
Intermediate	621 (26.1)	3.94 (3.90, 3.99)	
Junior	1,638 (68.6)	3.89 (3.86, 3.92)	
Workplace size			
Small	220 (9.2)	3.92 (3.83, 4.01)	0.1811
Medium	1,258 (52.9)	3.92 (3.88, 3.95)	
Large	902 (32.9)	3.88 (3.84, 3.92)	
Personal influence and development			
Low	1,063 (44.7)	3.61 (3.57, 3.65)	< 0.001
High	1,316 (55.3)	4.14 (4.11, 4.17)	
Interpersonal relationships and leadership			
Low	1,112 (46.8)	3.56 (3.53, 3.60)	< 0.001
High	1,266 (53.2)	4.20 (4.18, 4.23)	
Work–life balance			
Low	844 (35.5)	3.73 (3.69, 3.77)	< 0.001
High	1,536 (64.5)	4.00 (3.97, 4.03)	
Work values			
Low	986 (41.4)	3.44 (3.41, 3.48)	< 0.001
High	1,393 (58.6)	4.23 (4.20, 4.25)	
Respect for authorities			
Low	1,129 (47.5)	3.75 (3.71, 3.79)	< 0.001
High	1,250 (52.5)	4.04 (4.01, 4.07)	
Altruism			
Low	1,166 (49.0)	3.74 (3.71, 3.78)	< 0.001
High	1,213 (51.0)	4.06 (4.02, 4.09)	
Acceptance of authorities			
Low	860 (36.1)	3.95 (3.90, 3.99)	0.0049
High	1,520 (63.9)	3.88 (3.85, 3.91)	
*Mianzi* rule			
Low	822 (34.5)	3.86 (3.82, 3.90)	0.0087
High	1,558 (65.5)	3.93 (3.90, 3.96)	

Univariate analyses showed that older age and more working years were associated with lower WSC (Table 1). With the exception of workplace size, high levels of PWEs were positively associated with a high level of WSC. For example, participants with high perceived work value had significantly higher WSC scores (mean: 4.23; 95%CI: 4.20, 4.25) than those with low perceived work value (mean: 3.44; 95%CI: 3.41, 3.48). As for workplace TCVs, participants with high AA perceived lower WSC (mean: 3.88; 95%CI: 3.85, 3.91) than their counterparts with low AA (mean: 3.95; 95%CI: 3.90, 3.99). However, high RA, TA, and MR were positively correlated with high WSC.

The results of the multilevel ordinal regression models are shown in Table 2. The empty model (not shown in Table 2) indicated that there was significant variation in WSC across workplaces (*χ*^*2*^ = 215.16, *p* < 0.001); the interclass correlation coefficient (ICC) was 0.088, indicating that 8.8% of the variance in WSC was explained by a random effect for workplaces.

**Table 2:** The unstandardized coefficients and 95% confidence intervals of multilevel ordinal regression models for workplace social capital-associated individual- and workplace-level variables

	Model 1	Model 2	Model 3
Individual level variables			
Gender			
Male		0	0
Female		0.068 (− 0.093, 0.229)	0.058 (− 0.104, 0.221)
Age			
< 30		0	0
30–39		0.033 (− 0.183, 0.249)	0.007 (− 0.211, 0.225)
40–49		− 0.272 (− 0.540, − 0.003)	− 0.313 (− 0.584, − 0.042)
≥ 50		− 0.125 (− 0.462, 0.212)	− 0.155 (− 0.495, 0.184)
Educational level			
Junior high school		0	0
Senior high school		0.260 (0.047,0.472)	0.260 (0.047, 0.473)
Junior college		0.426 (0.179, 0.673)	0.435 (0.186, 0.683)
College or higher		0.365 (0.082,0.647)	0.403 (0.116, 0.691)
Marriage status			
Married		0	0
Unmarried		−0.024 (−0.237, 0.189)	−0.040 (− 0.253,0.173)
Annual salary (thousand yuan)			
< 50		0	0
50–100		−0.0001 (− 0.177, 0.178)	− 0.006 (− 0.185, 0.172)
≥ 100		−0.035 (− 0.343, 0.274)	− 0.041 (− 0.352, 0.270)
Working years			
< 2		0	0
2–3		−0.414 (−0.696, − 0.132)	− 0.441 (− 0.724, − 0.157)
4–5		−0.593 (− 0.903, − 0.282)	−0.618 (− 0.930, − 0.306)
6–7		−0.418 (− 0.760, − 0.077)	−0.444 (− 0.786, − 0.101)
8–9		−0.685 (−1.038, − 0.333)	− 0.704 (−1.059, − 0.348)
≥ 10		−0.622 (− 0.921, − 0.323)	−0.641 (− 0.942, − 0.339)
Position			
Senior		0	0
Medium		−0.083 (−0.443, 0.277)	−0.072 (− 0.433, 0.289)
Junior		−0.053 (− 0.402, 0.296)	− 0.038 (− 0.388, 0.312)
Personal influence and development			
Low		0	0
High		0.624 (0.446, 0.802)	0.626 (0.447, 0.805)
Interpersonal relations and leadership			
Low		0	0
High		1.071 (0.883, 1.256)	1.060 (0.870, 1.250)
Work–life balance			
Low		0	0
High		0.309 (0.144, 0.473)	0.280 (0.114, 0.447)
Work values			
Low		0	0
High		2.474 (2.259, 2.689)	2.467 (2.251, 2.683)
Respect to authorities			
Low		0	0
High		0.334 (0.144, 0.524)	0.325 (0.134, 0.516)
Altruism			
Low		0	0
High		0.357 (0.165, 0.549)	0.347 (0.155, 0.539)
Acceptance of authorities			
Low		0	0
High		−0.206 (−0.372, −0.040)	−0.214 (−0.381, −0.046)
*Mianzi* rule			
Low		0	0
High		−0.261 (− 0.438, − 0.084)	−0.258 (− 0.435, − 0.080)
Workplace-level variables			
Workplace size			
Small	0		0
Medium	−0.019 (− 0.468, 0.431)		0.114 (−0.310, 0.539)
Large	−0.247 (− 0.787, 0.293)		0.205 (−0.303, 0.713)
Personal influence and development			
Low	0		0
High	0.278 (−0.156, 0.712)		−0.182 (− 0.596, 0.232)
Interpersonal relations and leadership			
Low	0		0
High	0.129 (−0.242, 0.500)		0.182 (−0.173, 0.536)
Work−life balance			
Low	0		0
High	0.208 (−0.105, 0.520)		0.141 (−0.150, 0.434)
Work values			
Low	0		0
High	0.332 (−0.102, 0.766)		0.370 (−0.035, 0.774)
Respect for authorities			
Low	0		0
High	−0.081 (−0.575, 0.413)		0.093 (−0.373, 0.559)
Altruism			
Low	0		0
High	0.082 (−0.475, 0.640)		−0.329 (− 0.844, 0.186)
Acceptance of authorities			
Low	0		0
High	0.007 (−0.299, 0.313)		−0.038 (− 0.322, 0.246)
*Mianzi* rule			
Low	0		0
High	0.387 (−0.111, 0.884)		0.154 (−0.310, 0.539)

The Model 1 indicated that there was no workplace-level variable (including PWEs and workplace size) associated with WSC (Table 2). The Model 2 including only individual-level variables, found that participants with senior high school, junior college and college or higher had lower level of WSC(B: − 0.260, − 0.426 and − 0.365 respectively) compared with participants with junior high school, working years in the company were negatively associated with WSC (Table 2). Compared with participants under 30 years of age, those aged 40–49 had lower WSC (B: − 0.272; 95%CI: − 0.540, − 0.003). After controlling for individual socioeconomic characteristics, all individual-level PWEs, RA, and TA (B ranged from 0.334 to 2.474) were positively associated with WSC, while high individual-level AA (B: − 0.206; 95%CI: − 0.372, − 0.040), and MR (B: − 0.261; 95%CI: − 0.438, − 0.084) were negatively associated with WSC. Finally, all individual- and workplace-level variables were entered into the Model 3 simultaneously (Table 2). The associations between individual socioeconomic characteristics and WSC didn’t change significantly. After controlling for individual socioeconomic characteristics, all individual-level PWEs, RA, and TA (B ranged from 0.325 to 2.467) were still positively associated with WSC, while high individual-level AA (B: − 0.214; 95%CI: − 0.381, − 0.046) and MR (B: − 0.258; 95%CI: − 0.435, − 0.080) were negatively associated with WSC; no workplace-level variable was still associated with WSC.

## Discussion

Since social capital is one of the social determinants of population health and health-related behaviors, and the workplace is one of the main sources of social capital [3], it is important to explore the determinants of workplace social capital. This study found that higher education, favorable psychosocial work environments were positively associated with WSC, working years in the company was negatively associated with WSC, while workplace Chinese Confucian values had mixed relationships with WSC.

According to Huang’s (2009) review, education not only inculcates knowledge and social responsibilities but also shapes values, such as reciprocity, respect, and trust. In the present study, we found that education level was positively associated with WSC, which is consistent with previous findings [19, 21, 49]. Because social capital is built over time, stable long-term employment is important for building WSC, which can be destroyed by the regular use of temporary employees [50]. However, we had an unexpected finding that a greater number of years spent working at a company was associated with lower WSC. This is partly consistent with a previous finding that higher proportion of temporary employees and higher employee turnover were associated with higher levels of WSC [26]. Social networks play a special role in a person’s opportunity for mobility and provide better access to job information [51]. In China, nearly 61% of workers use social networks to search for work [52], and strong ties are frequently used [53]. In other words, new employees are social capitalists [26], who may gain the job in the current workplace by the social networks in the current workplace, and old employees with abundant social capital may switch to other companies.

An interesting finding of the present study was the favorable psychosocial work environments were positively associated with WSC. Previous studies also found that organizational justice, quality leadership and open communication [35, 36, 54] were positively associated with WSC. A previous randomized intervention study among Chinese community health (CHC) centers [55] indicated that team-building courses for CHC directors (team management, communication skills, and practical team leadership experiences) and group psychological consultations (on topics including team communications and stress management) for CHC staff can improve workplace social capital. There are at least two explanations for the associations between PWEs and WSC. First, favorable PWEs—such as higher personal influence and possibilities of development, open communication and role clarity, better interpersonal relationships and leadership, and higher work–life balance—may build harmonious social interactions and social cohesion [36, 56] and promote reciprocity, respect, and trust among employees, which are associated with bonding social capital. Second, quality leadership and organizational justice may promote open communication and connections across employees and employers to achieve common goals, and promote employees’ trust for employers and managers, which is associated with bridging social capital.

According to Putnam, social capital “refers to the collective value of all ‘social networks’ and the inclinations that arise from these networks to do things for each other [57].” Cultural context may influence social capital. In Asian countries, for example, the Chinese have very high levels of general trust and trust in the social system, while the Japanese have high general trust but very low trust in the social system [58]. Although researchers have argued that social capital is similar to *guanxi*, which is one of the main traditional values in China [41, 42], in this study, we found the surprising result that traditional Chinese workplace values have both positive and negative effects on WSC. Respect for authority (RA) and altruism (TA) were positively associated with WSC, while acceptance of authority and the *mianzi* rule were negatively associated with WSC. Workplace Chinese Confucian values aim to create harmonious interpersonal relationships, so employees may accept authorities’ (employers’ and managers’) decisions and demands, even when those are bad for the individual [46]. The *mianzi* rule [59] involves employees taking care of the social reputations of themselves, colleagues, and leaders to maintain and promote their social reputation in a variety of ways; this may require employees to do some things they do not want to do, such as accepting authorities’ decisions. Thus, acceptance of authorities and the *mianzi* rule do not constitute real trust, only superficial social manners, which may deteriorate social capital in the workplace.

### Limitations

Several limitations of this study should be noted. First, as is inherent in any cross-sectional study, the ability to draw causal inferences from the associations of workplace psychosocial factors and traditional Chinese workplace values with workplace social capital is substantially limited. Although we attempted to control for confounders, we cannot be certain that we have controlled for all possible confounders. For example, [26] found that workplace social capital was negatively associated with a high proportion of manual and male employees, job demand, which were not included in this study. Second, we tried to include a variety of workplaces, but this is not a representative sample incurring by selection bias, which may weaken both internal and external validity. We also could not survey employees who had resigned, whose status—including PWEs, workplace CCVs, and workplace social capital—may be different from that of the participants we surveyed in this study. Third, workplace social capital may be affected by factors outside workplaces that we did not assess in this study. For example, we found that high work–life balance was positively associated with workplace social capital, which suggests that family and personal life factors may affect workplace social capital. Longitudinal studies investigating the links between varied workplace psychosocial factors and workplace social capital among workers from varied industries would be worth conducting in the future.

## Conclusion

Despite some limitations, to the best of our knowledge, this is the first study to examine the associations between psychosocial work environments and workplace social capital in a Chinese context. Our study provides additional evidence that favorable psychosocial work environments—including personal development, leadership, work–life balance, and work value—is positively associated with workplace social capital, while workplace Chinese Confucian values have both positive and negative associations with workplace social capital. These findings suggest that workplace social capital associates with multiple factors. Psychosocial work environments and cultural context are important in understanding variations in workplace social capital between individuals.

## Abbreviations

AA: Acceptance of authorities
AL: Altruism
CCVs: Chinese Confucian values
CHC: Chinese community health
COPSOQ II: The second short version of the Copenhagen Psychosocial Questionnaire
MR: *Mianzi* rule
PWEs: Psychosocial work environments
RA: Respect for authorities
SES: Socioeconomic status
WSC: Workplace social capital
